# Literary Note

**Published:** 1911-06

**Authors:** 


### LITERARY NOTE

A Booklet of Special Value.—"Collargolum in urology, dermatology and radiology of the urinary tract" is the title of an attractive booklet just published by Schering & Glatz, 150-152 Maiden Lane, New York. It contains in compact form up-to-date information of interest to every physician engaged in genitourinary, dermatological or *x*-ray work. A copy will be mailed upon request. A limited edition only has been printed.

